# Head arteries of the red squirrel (*Sciurus vulgaris*)

**DOI:** 10.1007/s11259-022-10033-6

**Published:** 2022-11-10

**Authors:** Maciej Zdun, Jakub J. Ruszkowski, Mateusz Hetman, Mariusz Z. Felsmann

**Affiliations:** 1 Department of Animal Anatomy, Poznan University of Life Sciences, Wojska Polskiego 71C, 60-625 Poznań, Poland; 2 Department of Basic and Preclinical Sciences, Nicolaus Copernicus University in Torun, Lwowska 1, 87-100 Torun, Poland

**Keywords:** Sciuridae, Head vessels, Head vascularization, Angiology

## Abstract

The red squirrel *(Sciurus vulgaris)* is a medium-sized rodent protected in most of Europe. The present study aimed to investigate and describe the arterial vascularization of the head of the adult red squirrel. In the study, 48 specimens of adult red squirrels were used. The first preparation method used in the study was corrosion casting using a stained solution of the chemo-setting acrylic material injected into bilateral common carotid arteries resulting in corrosion castings of the vessels on a bone scaffold. The second method was injecting liquid-stained latex into both common carotid arteries. It resulted in a stained arterial vessel on fixed soft tissue preparations. The main vessels providing blood to the head were paired with common carotid arteries that divide into external and internal carotid arteries. The internal carotid artery passes into the stapedial artery. After giving its branches, the stapedial artery exits the cranial cavity through the sphenofrontal foramen and enters the orbit. The suborbital and the mandibular regions were supplied by the maxillary artery, linguofacial trunk, and their branches. Description of the detailed anatomy of the head arteries in red squirrels may contribute to establishing diagnostic and treatment protocols for wildlife rehabilitation centers, which may be crucial since red squirrels are endangered by the spreading of invasive Eastern gray squirrels (*Sciurus carolinensis*) in Europe. It may also contribute to veterinary care for other members of the Sciuridae family kept as pets.

## Introduction

The order Rodentia is the largest and most diversified among mammalian groups. Approximately 40% of mammals represent the order Rodentia (Nowak and Walker 1999). Sciuromorpha ("squirrel-like" rodents) is one of the three suborders of Rodentia (Capello 2005). Eurasian Red Squirrel *(Sciurus vulgaris)* is an arboreal, herbivorous rodent living in Europe and Asia. In 2006, it was assessed for The IUCN Red List of Threatened Species and listed as Least Concern species (Shar et al. 2016). The head arteries of the red squirrel have not been researched before. Aydin has described the morphology of circulus arteriosus cerebri and the arteries originating from the aortic arch and the branches of these arteries in this species (Aydin 2008, 2011). The arterial pattern of the head has been reported previously in other rodents – rats (Greene 1968), Botta's pocket gopher, Desmarest's spiny pocket mouse, spiny pocket mouse, long-tailed spiny pocket mouse, and kangaroo rats (Brylski 1990).

The red squirrel is protected by law in Poland and most of Europe. Qualified wildlife rehabilitation centers provide veterinary care for the species. Among diseases of the head region in squirrels, there have been reports of overgrown cheek teeth and overgrown and maloccluded incisors (Mancinelli and Capello 2016; Sainsbury et al. 2004), head trauma (Martínez-Jiménez et al. 2011), dermatophylosis (Holmes et al. 2019) and atypical histiocytosis of uncertain origin (Smith et al. 2017).

Medicine of other species from the Sciuridae family kept as pets, e.g., Eastern fox squirrel *(Sciurus niger),* is increasingly progressing, and specialistic case reports regarding advanced clinical techniques in those species are being published (Smith et al. 2013).

The present study aimed to name arterial vessels supplying blood to the head, investigate and describe arterial vascularization of the head of adult red squirrels of both genders and compare the results with that of other mammalian species described in scientific literature. The detailed anatomy of the head arteries in red squirrels may contribute to establishing diagnostic and treatment protocols, improving surgical techniques and avoiding complications (e.g., hemorrhage). It may be more significant in the future since red squirrels are endangered by spreading the invasive Eastern gray squirrel *(Sciurus carolinensis)* in Europe (Stevenson-Holt and Sinclair 2015; Signorile et al. 2014). The presented results may also be a base for further anatomical studies, including vascular anatomy on other species belonging to the Sciuridae family.

## Materials and methods

### Animals

Therefore red squirrels are protected by Polish law (Regulation of the Minister of the Environment of 16 December 2016, on the protection of animal species (Journal of Laws, item 2183) and (Journal of Laws 2020, item 26), all procedures done to accomplish the goal of the study were approved and carried out following the appropriate regulations and permits (Regional Directorate for Environmental Protection in Poznan (Poland): WPN-II.6401.366.2020.TE).

The study was conducted on preparations gathered in the Department of Animal Anatomy of Poznań University of Life Sciences (Poland). In the study, preparations from adult animals of both genders are included.

### Methods

Thirty-five randomly selected cadavers were processed by injecting a COLOREX® (Śnieżka, Poland) stained solution of the chemo-setting acrylic material Duracryl® Plus (SpofaDental, Czech Republic) into both the common carotid arteries. After a short time (15–20 min) necessary for setting, the specimens were enzymatically macerated with Persil® powder (Henkel, Germany) and diluted in water at 42 °C for 15 days. This procedure resulted in corrosion castings of the vessels on a bone scaffold (without the animal's tissues, except the bones). The second method, applied to 13 specimens, consisted of passing the liquid-stained latex LBS 3060 (Synthos, Poland) into both common carotid arteries, leaving it to set in a 5% formalin solution (Chempur, Poland) for two weeks, then preparing the blood vessels manually using surgical instruments during dissection, to view them within the tissue.

The names of the anatomical structures were standardized according to Nomina Anatomica Veterinaria and Wahlert's work. (International Committee on Veterinary Gross Anatomical Nomenclature 2017; Wahlert 1985).

## Results

**Fig. 1:**
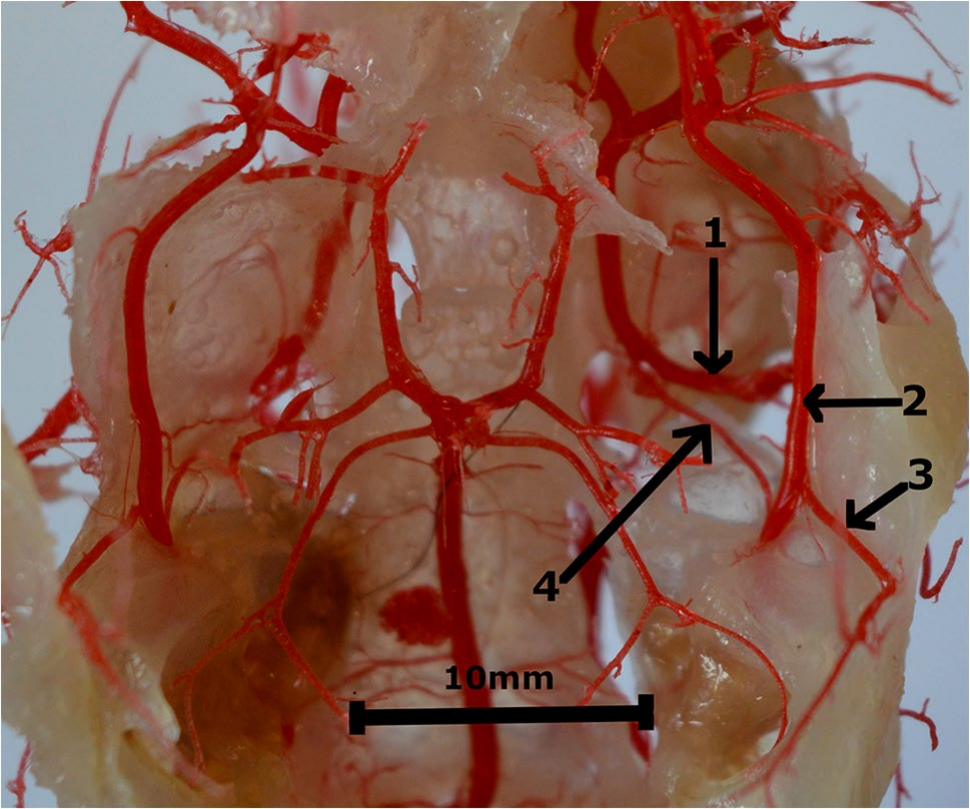
Arteries in the cranial cavity. Corrosion cast. 1 – the maxillary artery. 2 – the stapedial artery. 3 – the middle meningeal artery. 4 – the anastomosing branch from the stapedial artery to the maxillary artery

Blood to the head is provided by the bilateral common carotid arteries *(arteriae carotes communes)*. Each side artery is divided into the external carotid artery *(arteria carotis externa)* and the internal carotid artery *(arteria carotis interna)*. The internal carotid artery is a strong vessel that forms a common trunk with the occipital artery *(arteria occipitalis)* and ascending pharyngeal artery *(arteria pharyngea ascendens).* The last of the above-mentioned vessels branched into the muscles of the pharynx. The occipital artery lies on the squamous part of the occipital bone supplying the muscles in the area. The internal carotid artery passes into the stapedial artery *(arteria stapedia)* (Fig. 1). Another fragment of the internal carotid artery is not present. After passing through the tympanic cavity, the stapedial artery lays on the lateral surface of the cranial cavity. From the stapedial artery branched off the anastomosing branch to the maxillary artery *(arteria maxillaris)*, which exits the cranial cavity through the foramen ovale *(foramen ovale)*. Near, branched off the medial meningeal artery *(arteria meningea media)*, which extends in the caudal deep temporal artery *(arteria temporalis profunda caudalis)*. This vessel exits the cranial cavity through the temporal meatus *(meatus temporalis)*.

**Fig. 2:**
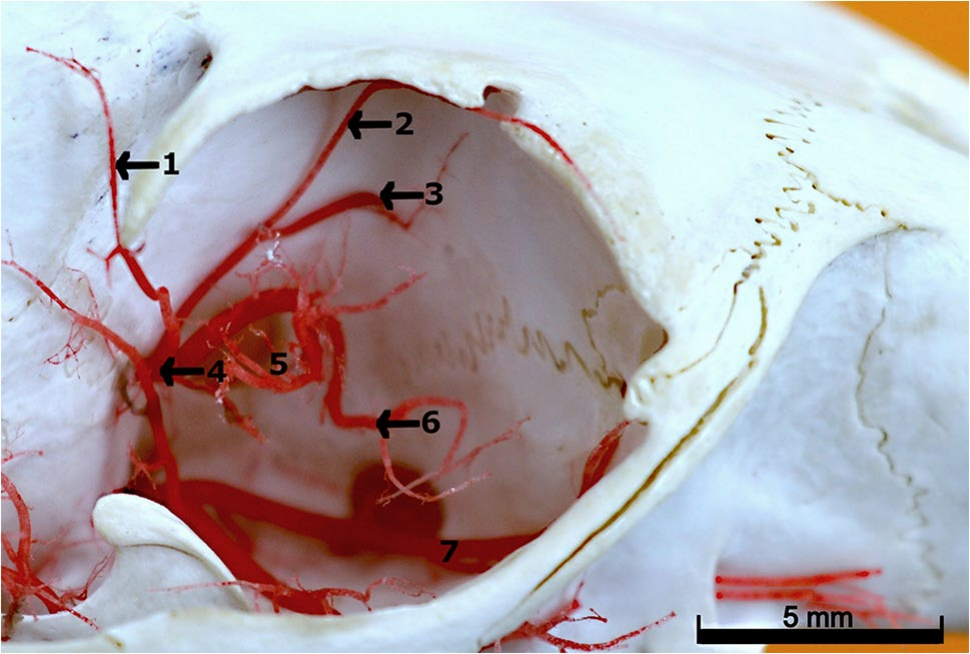
Arteries in the orbit. Corrosion cast. 1 – the rostral deep temporal artery. 2 – the supraorbital artery. 3 – the ethmoidal artery. 4 – the rostral deep temporal artery. 5 – the ciliary arteries. 6 – branch to the muscles of the eyeball. 7 – the maxillary artery

Next, the stapedial artery exits the cranial cavity through the sphenofrontal foramen and enters the orbit. In the orbit, it is joined by a fragile internal ophthalmic artery *(arteria ophthalmica interna)* which passes through the optic canal *(canalis opticus)*. From the stapedial artery branched off the external ethmoidal artery *(arteria ethmodalis externa)* (Fig. 2). From this vessel branched off the rostral deep temporal artery *(arteria temporalis profunda rostralis)* and supraorbital artery *(arteria supraorbitalis)*. Next, from the stapedial artery branched off the ciliary arteries *(arteriae ciliares),* after which this vessel eventually heads to the muscles that move the eyeball.

**Fig. 3:**
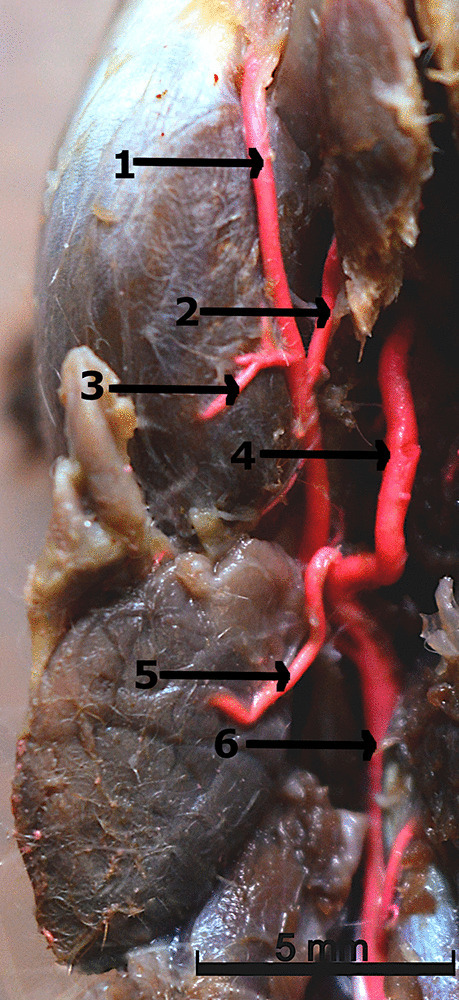
Latex preparation of the intermandibular region. 1 – the facial artery. 2 – the sublingual artery. 3 – the masseteric branch of the facial artery. 4 – the lingual artery. 5 – the glandular branch of the facial artery. 6 – the linguofacial trunk

A vessel with a much larger diameter than the internal carotid artery is the external carotid artery. At the point where the linguofacial trunk *(truncus linguofacialis)* branched off from the external carotid artery (Fig. 3), a reduction in the diameter of the main arterial trunk was observed. At this point, two vessels of equal diameter were formed. The first was the linguofacial trunk, and the second was the continuation of the external carotid artery. The linguofacial trunk is a short vessel divided into the lingual artery *(arteria lingualis)* and the facial artery *(arteria facialis)*. The lingual artery is a strong vessel from which the submental artery *(arteria submentalis)* branched off. Then, the lingual artery evolves into a deep lingual artery *(arteria profunda linguae)* with dorsal lingual branches *(rami dorsales linguae)*. The facial artery is a weak vessel. The sublingual artery *(arteria sublingualis)* branched off from them.

Moreover, branches to the pterygoid muscles, masseter muscle, and glandular branches to the mandibular salivary gland branched off. The facial artery wraps around the edge of the mandible and lies on the facial surface of the mandible. The site of this wrapping is in the 1/4 of the rostral part of the corpus, close to the incisor part of the mandibular corpus. Then, the inferior labial artery *(arteria labialis inferior)*, the angular oral artery *(arteria angularis oris),* and the superior labial artery *(arteria labialis superior)* branched off. The superior labial artery is a stronger vessel than the inferior one.

**Fig. 4:**
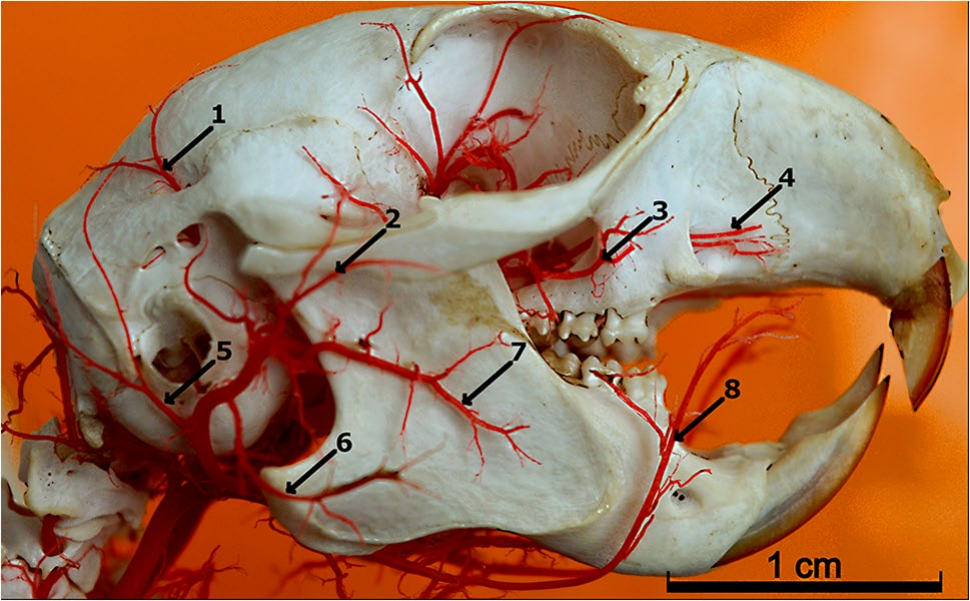
The facial surface of the face. Corrosion cast. 1 – the caudal deep temporal artery. 2 – the superficial temporal artery. 3 – the buccal artery. 4 – the infraorbital artery. 5 – the caudal auricular artery. 6 – the masseteric branch. 7 – the transverse facial artery. 8 – the facial artery

After the linguofacial trunk branched off, the external carotid artery is directed dorsally. The caudal auricular artery *(arteria auricularis caudalis)* branched off (Fig. 4). It is a small vessel divided into two branches. Then, the branch to the masseter muscle *(ramus massetericus)* branched off. This is a strong vessel that lies superficially. Close to it, the transverse facial artery *(arteria transversa facialis)* branched off. This strong vessel heads to the masseter muscle. From the transverse facial artery branched off the superficial temporal artery *(arteria temporalis superficialis)*, rostral auricular artery *(arteria auricularis superficialis),* and muscular branch. Due to the diameter of the vessels, it was treated that the superficial temporal artery branched off from the transverse facial artery and not vice versa. In 8 specimens, the transverse facial artery diverged independently from the main arterial trunk, while the other two vessels branched off with a common trunk from the external carotid artery.

**Fig. 5:**
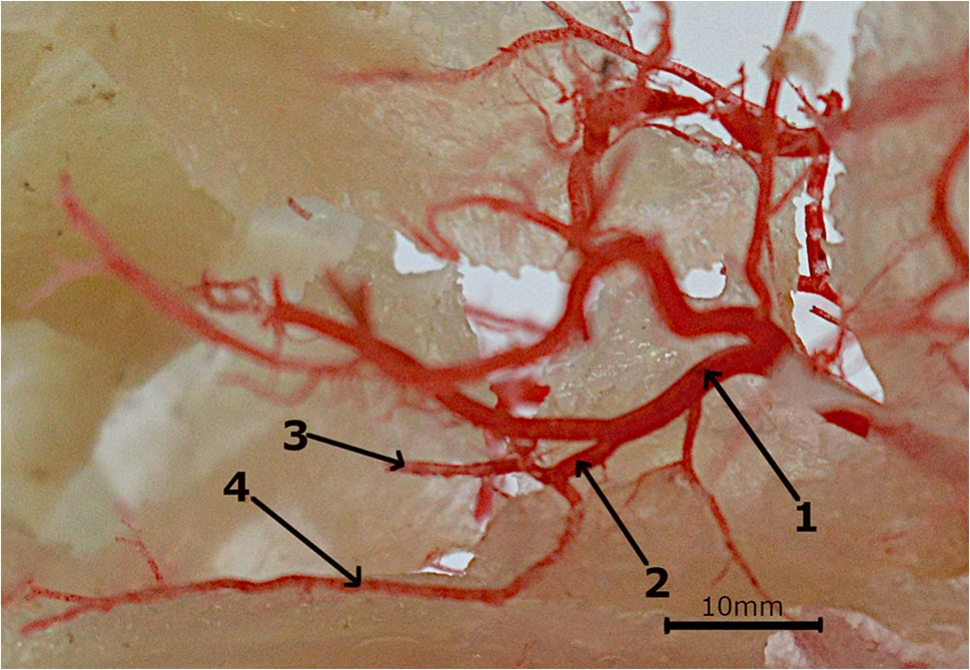
Some terminal branches of the maxillary artery. Corrosion cast. 1 – the maxillary artery. 2 – the descending palatine artery. 3 – the sphenopalatine artery. 4 – the major palatine artery

After branching off this last vessel, the main arterial stream of the head is renamed into the maxillary artery. As the first, the inferior alveolar artery *(arteria alveolaris inferior)* and the rostral deep temporal artery branched off. The inferior alveolar artery runs in the mandible canal, heading for the dental alveoli. Then, the maxillary artery penetrates the caudal alar foramen and leaves the rostral alar foramen lying on the bottom of the orbit. In this place branched off the buccal artery *(arteria buccalis)*. This vessel arranges more laterally and supplies the cheek. Next, the malar artery *(arteria malaris),* which supply the third palpebra, and the area of the medial angle of the eye branched off from the maxillary artery. As a result of the final division branched off the minor palatine artery *(arteria palatina minor)*, the descending palatine artery *(arteria palatina descendens),* and the infraorbital artery *(arteria infraorbitalis)*. The minor palatine artery vascularizes the soft palate. The descending palatine artery is divided into the major palatine artery *(arteria palatina major),* which supplies the hard palate, and the sphenopalatine artery *(arteria sphenopalatina)*, supplying the nasal cavity (Fig. 5). The infraorbital artery penetrates the infraorbital canal where the dental branches *(rami dentales)* branched off. The rostral section supplies the lateral and dorsal parts of the nose as the dorsal nasal artery *(arteria dorsalis nasi)* and lateral nasal artery *(arteria lateralis nasi).* In five specimens, the rostral part of the infraorbital artery was double.

## Discussion

In our study, the stapedial artery was found in the red squirrel. Bugge (1970, 1971a, b) distinguished three branches of this artery: supraorbital, infraorbital, and mandibular branches. All these branches were observed in hamsters, grasshopper mice, the deer mouse (Bugge 1970), garden dormouse, African dormouse (Bugge 1971a), red squirrel, yellow-handed tree squirrel, long-nosed squirrel, giant flying squirrel, Eastern flying squirrel, Indo-Malaysian flying squirrel, prince desert pocker mouse, Bailey's pocket mouse, Merriam's kangaroo rat, Western banner-tailed kangaroo rat (Bugge 1971c). This information, as far as the red squirrel is concerned, contradicts our observations. Our study shows that only the derivatives of the supraorbital branch originate from the stapedial artery. The vessels heading to the suborbital and mandibular regions are derivatives of the maxillary artery. Bugge's study was conducted on only four individuals of the red squirrel. However, in our study, we did not find a single individual with the model described by Bugge (1971c). In other species, it has been described that some of the branches mentioned above were not present. In voles, striped field mice, long-tailed field mice, and yellow-necked field mice, the infraorbital and mandible branches were described (Bugge 1970). In lemming, gerbils, and some murids, only the infraorbital branch is present. Other vessels branched off from the internal–external carotid system (Bugge 1970). The absence of a stapedial artery has been described in some rodents, such as beavers, mountain beavers, Eastern American pocket gophers (Bugge 1971c), Hystricidae, Erethizontidae, Caviidae, Dasyproctidae, chinchillas, nutrias, degu, cane rats (Bugge 1971b), mole-rat, bamboo rats, dormouse (Bugge 1971a).

The presence of the strong and well-developed internal carotid artery was found in agouti (Silva et al. 2016), Mongolian gerbil (Kuchinka et al. 2008), beaver (Bugge 1971c; Frąckowiak and Śmiełowski 1998), rat (Greene 1968), prince desert pocket mouse, Bailey's pocket mouse, Merriam's kangaroo rat, Western banner-tailed kangaroo rat (Bugge 1971c), mole-rat, bamboo rats (Bugge 1971a), Erethizontidae (Bugge 1971b). In species where the stapedial artery is present and partial obliteration of the internal carotid artery occurs, the initial section of the internal carotid artery is preserved, extending into the stapedial artery. Such a vascular pattern has been found in our studies, as well as in Dolan's spring ground squirrel, grey-tailed antelope squirrel, Eastern American chipmunk, Asiatic chipmunk, marmot, red squirrel, palm squirrel, yellow-handed tree squirrel, long-nosed squirrel, giant flying squirrel, Eastern flying squirrel, Indo-Malaysian flying squirrel (Bugge 1971c), garden dormouse, African dormouse (Bugge 1971a).

Popesko et al. (2010) observed the linguofacial trunk in rats, but Greene (1968) reported that the lingual artery originates from the external carotid artery. The presence of a linguofacial trunk has also been described in Equidae (Kowalczyk and Frąckowiak 2017), Moschus, Cervidae, Aepycerotinae, Alcelaphinae, Bovinae, Hippotraginae, Reduncinae and some part of the Antilopinae from the Ruminantia (Zdun et al. 2014). Without the linguofacial trunk, the lingual artery branched off from the external carotid artery. The same is true of the facial artery, although some differences exist. The facial artery branched off from the external carotid artery in the rhinos, tapirs (Kowalczyk and Frąckowiak 2017), Suinae, peccary, and hippos (Kowalczyk and Frąckowiak 2019), Giraffa, Rangifer (Zdun et al. 2014), Botta's pocket gopher, Desmarest's spiny pocket mouse, spiny pocket mouse, long-tailed spiny pocket mouse, kangaroo rats (Brylski 1990) and rat (Greene 1968). In camels and llamas, the facial artery branched off by the common trunk with the caudal auricular artery (Kowalczyk et al. 2018). In the Caprinae, Saiga, and Springbok representatives, there is no facial artery (Zdun et al. 2014). The facial artery gives off the glandular branch, masseteric branches, and submental artery in rats (Greene 1968), ruminants (Zdun et al. 2014), and rhinos (Kowalczyk and Frąckowiak 2017). In the Equidae submental artery branched off from the sublingual artery. In the tapirs, the submental artery branched off from the inferior alveolar artery (Kowalczyk and Frąckowiak 2017). In hamsters, the submental artery branched off from the facial artery as the sublingual artery (Popesko et al. 2010). Except for the Equidae and hamsters, the sublingual artery branched off from the facial artery in mice (Popesko et al. 2010). Then, the facial artery moves to the facial surface of the mandible near the notch for facial vessels in ruminants (Zdun et al. 2014), Equidae, tapirs, and rhinos (Kowalczyk and Frąckowiak 2017) or in close to the angle of the mandible in Suidae (Kowalczyk and Frąckowiak 2019). In camels and llamas, this vessel moves on the mandible's caudal edge of the ramus (Kowalczyk et al. 2018). Our study found that the passage point was located more rostrally from the notch for facial vessels. In Suidae, this vessel is weak and ends in the masseter muscle (Kowalczyk and Frąckowiak 2019). In rhinos, tapirs, camels, and llamas inferior labial artery and angular artery of the mouth branched off from the facial artery Kowalczyk and Frąckowiak 2017; Kowalczyk et al. 2018). In Equidae, rats, mice, and hamsters, this vessel is developed much more strongly, branching off the superior labial artery and supplying the nose (Greene 1968; Kowalczyk and Frąckowiak 2017; Popesko et al. 2010). In some species, such as Desmarest's spiny pocket mouse, spiny pocket mouse, long-tailed spiny pocket mouse, and kangaroo rats, the transverse facial artery and the superficial temporal artery branched off by a common trunk. The masseteric artery branched off independently (Brylski 1990). In Botta's pocket gopher all three arteries (the transverse facial artery, the superficial temporal artery, and the masseteric artery) branched off together by a common trunk (Brylski 1990), while in the rat, all these vessels branched off independently, from the external carotid artery, without forming a common trunk (Greene 1968). In the llamas, the transverse facial artery and the superficial temporal artery create a common trunk with the inferior alveolar artery. In contrast, in camels, this common trunk also contains the deep temporal artery (Kowalczyk et al. 2018). In domestic mammals, the transverse facial artery branched off from the superficial temporal artery (Nickel and Schwarz 1963; Popesko et al. 2010). In most species, the inferior alveolar artery branched off from the main arterial trunk, while in Botta's pocket gopher, this vessel branched off together with the pterygoid artery (Brylski 1990; Greene 1968; Nickel and Schwarz 1963; Popesko et al. 2010). The ophthalmic artery in most species branched off from the maxillary artery, but in mice and hamsters branched off from the stapedial artery. In Botta's pocket gopher ophthalmic artery branched off together with the superior alveolar artery (Brylski 1990; Greene 1968; Nickel and Schwarz 1963; Popesko et al. 2010).

The arrangement of vessels supplying the palate corresponded to that of the dog (Hermanson et al. 2018) and cat (Nickel and Schwarz 1963). In other species, the minor palatine artery branched off from descending palatine artery (Nickel and Schwarz 1963). The arrangement of vessels supplying the palate corresponded to that of the dog.

The terminal branches of the main arterial stream include the infraorbital artery, which originates from the maxillary artery or the stapedial artery. It depends on whether the stapedial artery has an infraorbital branch or not, as we wrote at the beginning of the discussion. Similarly, the area of supply is expressed differently, depending on whether the facial artery participates in the vascularization of this area to a greater or lesser extent.

To sum up, there have been significant differences in the area of blood supply of the stapedial artery – it was smaller than described previously by Bugge (1971c). The arterial pattern described in our study is closest to the Eastern red squirrel and palm squirrel. The arteries supplying the maxilla and the mandible originate from the maxillary artery. These regions' vascularization patterns may be considered while planning surgical procedures.

## Data Availability

The data used to support the findings of this study are available from the corresponding author upon reasonable request.
